# Application of bulk segregant RNA-Seq (BSR-Seq) and allele-specific primers to study soybean powdery mildew resistance

**DOI:** 10.1186/s12870-024-04822-1

**Published:** 2024-03-01

**Authors:** Cheng-Chun Huang, Chen-Hsiang Lin, Yu-Cheng Lin, Hao-Xun Chang

**Affiliations:** 1 Master Program for Plant Medicine, National Taiwan University, Taipei, 106319 Taiwan; 2 Taoyuan District Agricultural Research and Extension Station. Ministry of Agriculture, Taoyuan, 327005 Taiwan; 3 Department of Plant Pathology and Microbiology, National Taiwan University, Taipei, 106319 Taiwan; 4 Present Address: Department of Ecology and Evolutionary Biology, The University of Michigan, Ann Arbor, MI 48109 USA; 5 Center of Biotechnology, National Taiwan University, Taipei, 106319 Taiwan

**Keywords:** Soybean, Powdery mildew, Bulk segregant RNA-Seq, MLO gene, QTL mapping, Transcriptome, Resistance

## Abstract

**Background:**

Powdery mildew (PM) is one of the important soybean diseases, and host resistance could practically contribute to soybean PM management. To date, only the *Rmd* locus on chromosome (Chr) 16 was identified through traditional QTL mapping and GWAS, and it remains unclear if the bulk segregant RNA-Seq (BSR-Seq) methodology is feasible to explore additional PM resistance that might exist in other varieties.

**Results:**

BSR-Seq was applied to contrast genotypes and gene expressions between the resistant bulk (R bulk) and the susceptible bulk (S bulk), as well as the parents. The ∆(SNP-index) and G’ value identified several QTL and significant SNPs/Indels on Chr06, Chr15, and Chr16. Differentially expressed genes (DEGs) located within these QTL were identified using HISAT2 and Kallisto, and allele-specific primers (AS-primers) were designed to validate the accuracy of phenotypic prediction. While the AS-primers on Chr06 or Chr15 cannot distinguish the resistant and susceptible phenotypes, AS-primers on Chr16 exhibited 82% accuracy prediction with an additive effect, similar to the SSR marker Satt431.

**Conclusions:**

Evaluation of additional AS-primers in the linkage disequilibrium (LD) block on Chr16 further confirmed the resistant locus, derived from the resistant parental variety ‘Kaohsiung 11’ (‘KS11’), not only overlaps with the *Rmd* locus with unique up-regulated LRR genes (Glyma.16G213700 and Glyma.16G215100), but also harbors a down-regulated MLO gene (Glyma.16G145600). Accordingly, this study exemplified the feasibility of BSR-Seq in studying biotrophic disease resistance in soybean, and showed the genetic makeup of soybean variety ‘KS11’ comprising the *Rmd* locus and one MLO gene.

**Supplementary Information:**

The online version contains supplementary material available at 10.1186/s12870-024-04822-1.

## Background

Marker-assisted selection (MAS) has emerged as a successful strategy in plant breeding based on two advantages, which include the availability of cost-effective molecular markers and the association between the trait of interest and the markers [1, 2]. Traditionally, molecular markers such as restriction fragment length polymorphisms (RFLP), amplified fragment length polymorphisms (AFLP), and simple sequence repeats (SSR) were widely used for linkage maps and MAS. These markers have limitations in terms of low density and labor-intensive processes, and the reliance on recombination frequency often resulted in lower genomic resolution and large intervals associated with a trait [3, 4]. With the advent of high-throughput sequencing technologies, single nucleotide polymorphisms (SNPs) have become the predominant molecular markers for MAS. SNPs offer several advantages, including increased marker density and reduced labor and time costs. Genotyping-by-sequencing (GBS), restriction-site-associated DNA sequencing (RAD), and whole genome re-sequencing (WGRS) have made it convenient to obtain millions of SNPs with precise physical positions in plant genomes [5]. Additionally, the development of allele-specific primers (AS-primers) like Kompetitive allele-specific PCR (KASP) has sped up the detection of specific genotypes and SNPs. Moreover, fluorescent quantification in allele-specific PCR allows measurement of copy numbers, extending its advantages from qualitative detection to quantitative analysis of traits such as soybean *rhg1* loci associated with resistance to soybean cyst nematode [6, 7]. Accordingly, the advantages of SNPs as molecular markers have surpassed traditional markers, and their potential is actively being explored.

Regarding the association between molecular markers and the trait of interest, classic interval mapping or quantitative trait loci (QTL) mapping relies on genetic recombination in bi-parental crossing populations. However, the limited recombination may restrict mapping resolution, and the exploration of genetic backgrounds is restricted within the genetic backgrounds of parents. Alternative mapping strategies such as genome-wide association studies (GWAS) serve as a complementary method to interval mapping [8]. Unlike bi-parental populations, GWAS leverages natural diversity panels that represent a genetic reservoir of historical recombination events. By scanning associations among diverse germplasm collections, GWAS enables the discovery of resistance loci across multiple genetic backgrounds [9]. Moreover, high-density SNPs facilitate linkage disequilibrium (LD) analysis on the targeted regions, narrowing down the genomic region harboring candidate genes. Nevertheless, the performance of GWAS may vary depending on SNP density and quality, as well as the statistical models. Consequently, integration of GWAS with QTL mapping [10], synteny analysis [11], or differential expression analysis [12] has been utilized to provide parallel clues for identifying disease resistance genes in soybean.

Regardless of a bi-parental crossing population or a diversity panel, the cost of phenotyping the entire population presents a significant burden. As a result, a mapping strategy was proposed to focus on individuals with extreme phenotypes within a population, and the concept of bulking segregants was demonstrated to be effective in multiple cases [13, 14]. For instance, in studying Fusarium wilt resistance in pigeonpea, 16 resistant and 16 susceptible individuals were selected from a population of 188 recombinant inbred lines (RILs) for bulk segregant DNA sequencing (BSA-Seq) [15]. Another BSA-Seq study utilized four bulks from two RIL populations to identify resistance to Ascochyta blight in chickpea [16]. More recently, in a study comparing a population of 50 resistant and 50 susceptible F_2_ individuals of sorghum, BSA-Seq successfully identified the *ARG2* locus on chromosome (Chr) 5 associated with anthracnose resistance, which subsequently guided the discovery of ARG2-mediated resistance [17]. While these studies exemplify the feasibility of the BSA strategy in uncovering plant resistance to necrotrophic or hemibiotrophic pathogens, there are even more studies demonstrated the power of BSA in studying plant resistance to biotrophic pathogens such as rust and powdery mildew (PM) pathogens. For example, in studying wheat stripe rust, 30 resistant and 30 susceptible F_2:3_ lines were investigated, and bulk segregant exome capture sequencing (BSE-Seq) was performed by utilizing exome capture from the bulked DNA. The results identified the YrXH-1AL locus on Chr 1 A, and two KASP markers were developed for MAS [18]. Similarly, in the cases of maize southern corn rust and melon powdery mildew diseases, BSA-Seq was conducted on 25 resistant and 25 susceptible individuals from a BC_4_F_2_ maize population [19] and a F_2_ melon population [20], respectively. Notably, RNA-Seq was employed in these maize and melon studies to examine differentially expressed genes (DEGs) in the parental lines. The transcriptomics analyses provided additional insights to identify candidate resistance genes within the QTL regions. Collectively, these examples demonstrate not only the potential of BSA-Seq in studying disease resistances across various crops but also the advantage of integrating RNA-Seq transcriptomic data.

In order to leverage the benefits of BSA-Seq and RNA-Seq, a hybrid approach named bulk segregant RNA-Seq (BSR-Seq) was devised, involving the identification of SNPs from RNA sequences [21]. BSR-Seq differs from BSA-Seq in that it calls SNPs from mRNA, allowing the identification of candidate genes through not only SNPs analysis but also differential expression analysis by contrasting the extreme bulks. Although the SNPs calling is restricted to the 5’-UTR, exons, and 3’-UTR, BSR-Seq has demonstrated successful applications in studying various disease resistances. For instance, BSR-Seq was employed in the investigation of stem rust resistance, where 12 resistant and 11 susceptible F_3_ lines were pooled for analysis. This study successfully identified a locus on Chr 2U with two potential nucleotide-binding site and leucine-rich repeat (NLR) protein candidates [22]. Similarly, BSR-Seq was utilized in the examination of Aphanomyces root rot in pea, where a comparison between 25 resistant and 25 susceptible lines led to the identification of hundred SNPs located within 31 candidate genes for further investigation [23]. Furthermore, BSR-Seq has been applied to investigate clubroot resistance in canola using 30 plants per bulk [24], as well as barley for resistance against barley yellow mosaic virus (BaYMV) and barley mild mosaic virus (BaMMV) using 16 plants per bulk [25]. Both studies successfully identified significant QTLs, enabling the development of AS-primers for selection.

Moreover, BSR-Seq has been extensively employed in the study of wheat diseases such as stripe rust. For example, a population consisting of 50 resistant and 50 susceptible F_2:5_ lines were investigated, leading to the mapping of the Yr041133 locus on Chr 7B, with a leucine-rich repeat (LRR) receptor-like protein kinase gene (TraesCS7B01G352400) identified as a candidate gene [26]. On the other hand, BSR-Seq was applied to a population of 10 resistant and 10 susceptible F_2_ lines, resulting in the discovery of the YrCf75 locus, which was associated with resistance and linked to 31 KASP markers and one SSR marker [27]. In the case of wheat spot blotch disease, 30 resistant and 30 susceptible lines from the RIL population underwent BSR-Seq analysis, leading to the identification of disease-associated transcripts and SNPs on Chr 3B and 5B, which were subsequently utilized for the development of AS-primers [28].

In the specific case of wheat powdery mildew (PM) disease, a study utilized BSR-Seq to analyze a set of 20 resistant and 20 susceptible F_2:3_ lines. This analysis led to the identification of two significant regions on Chr 2B, within which 22 DEGs, including two LRR genes [29]. In addition, BSR-Seq was employed in another study with a population consisting of 50 resistant lines and 50 susceptible F_2:3_ lines. This investigation resulted in the development of 7 KASP markers for the resistant locus *PmCH7087* on Chr 2B [30]. In a separate study, the focus on the *PmPBDH* locus using 50 resistant and 50 susceptible F_2:3_ lines not only revealed candidate genes such as RPP13 resistance-like genes and LRR receptor-like kinase, but also facilitated the development of 2 KASP markers for MAS [31]. Another BSR-Seq study focused on the dominant *PmLS5082* locus on Chr 2BL arm by contrasting 50 resistant and 50 susceptible wheat lines. Within the locus interval, the study identified 6 candidate genes and developed 10 markers to facilitate MAS [32]. As for the *pmHYM* locus on Chr 7BL, a study utilized 50 resistant and 50 susceptible F_2:3_ lines to target this locus to a 12.95 Mb region. There were several candidate genes in this region, including the disease resistance protein RGA4, which exhibited higher expression in the resistant parent [33]. Lastly, BSR-Seq was applied to a population consisting of 30 resistant lines and 30 susceptible F_2:3_ homozygous lines, successfully identifying the *PmSN15218* locus on wheat Chr 2AL [34].

While numerous cases have demonstrated the efficacy of BSR-Seq in various plant systems, there has been no study thus far that validates the applicability of BSR-Seq in soybean. Multiple studies have focused on soybean phenotypes, such as crinkled leaf, isoflavones in seeds, male sterility, rolled leaf, and short petioles, using BSA-Seq. These studies then supported the mapping results with separate RNA-Seq or RT-qPCR to investigate the expression of candidate genes [35–38]. Although an RNA-Seq study on bulked samples was conducted for the four-seed-per-pod trait, the study did not evaluate the potential advantages of SNP calling on RNA [39]. Consequently, there remains a need for proof-of-concept to validate BSR-Seq in the soybean system.

This research presents two objectives. The first aim is to validate the feasibility of BSR-Seq in the soybean system. The second objective is to utilize BSR-Seq to uncover the PM (powdery mildew) resistance in a soybean variety from Taiwan. Since soybean PM is caused by a biotrophic fungus called *Microsphaera diffusa* [40], which is a biotrophic pathogen similar to the wheat PM and rust fungus, the application of BSR-Seq in exploring soybean PM resistance should be promising. Overall, this study successfully confirmed the feasibility of BSR-Seq and identified the *Rmd* locus and candidate genes in the soybean variety ‘KS11’.

## Results

### Inheritance of powdery mildew (PM) resistance derived from the soybean cultivar ‘KS 11’

**Fig. 1:**
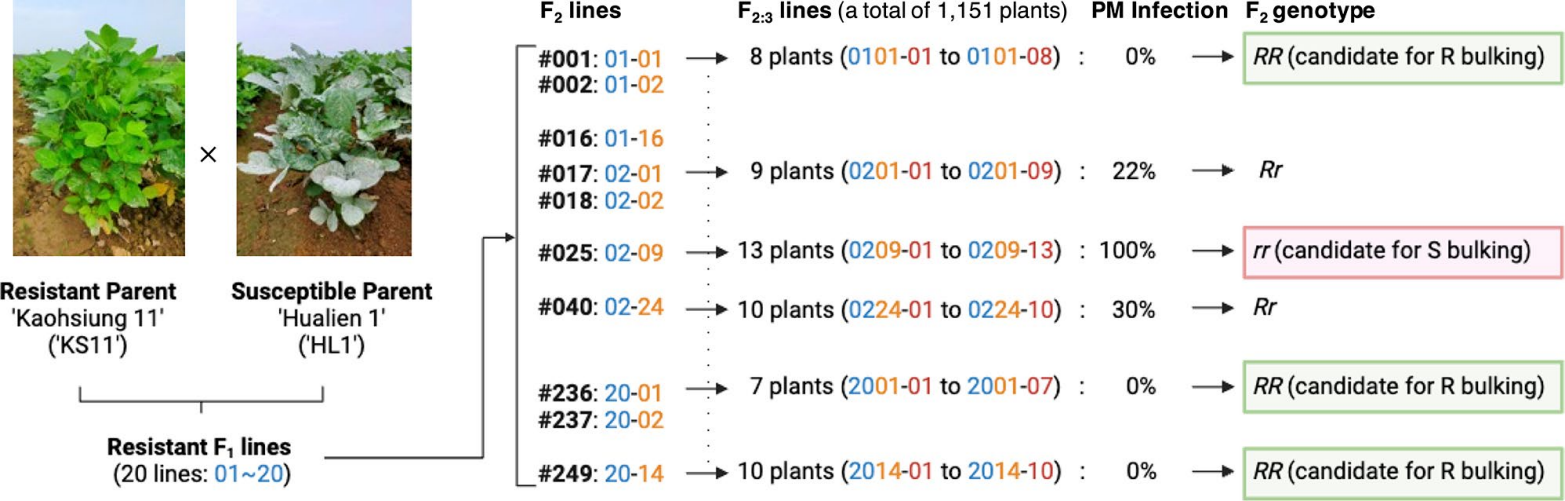
Schematic illustration for the ‘Kaohsiung 11’ x ‘Hualien 1’ crossing population and bulking. There were 20 F_1_ plants to generate 249 F_2_ plants. The F_2_ genotypes (*RR*, *Rr*, or *rr*) were determined based on the powdery mildew (PM) infection rate on F_2:3_ plants. Homozygous *RR* or *rr* would be bulked into R bulks and S bulks if the observed F_2:3_ lines were over 10 and 6 individuals, respectively. There were three R bulks and three S bulks as biological replicates, and each bulk contained ten F_2_ lineages. In other words, a total of 60 resistant F_2_ leaf RNA was compared to 60 susceptible F_2_ leaf RNA in the bulk segregant analysis

The dominant inheritance of PM resistance derived from ‘KS11’ was confirmed by observing a complete resistance in all F_1_ plants across the growth stages. To assess whether the resistance is monogenic, oligogenic, or polygenic, disease incidence was planned to be evaluated in the F_2_ population. The results revealed that among the F_2_ lineages, 153 displayed 0% PM infection, 54 exhibited PM infection ranging from 8–92%, and 42 showed 100% PM infection (Fig. 1). In other words, the F_2_ population included 153 and 54 lineages with genotypes presumed as homozygous resistance (*RR*) and heterozygous resistance (*Rr*), and 42 lineages with genotypes presumed as homozygous susceptibility (*rr*) (Supplementary Material 1). As the observed ratio of 207:42 significantly deviate from the expected Mendelian ratio of 3:1 (χ^2^, *p* = 0.528) based on the test for goodness of fit, the PM resistance of ‘KS11’ is considered to have more than one loci.

### Bulk segregant analysis (BSA)

**Fig. 2:**
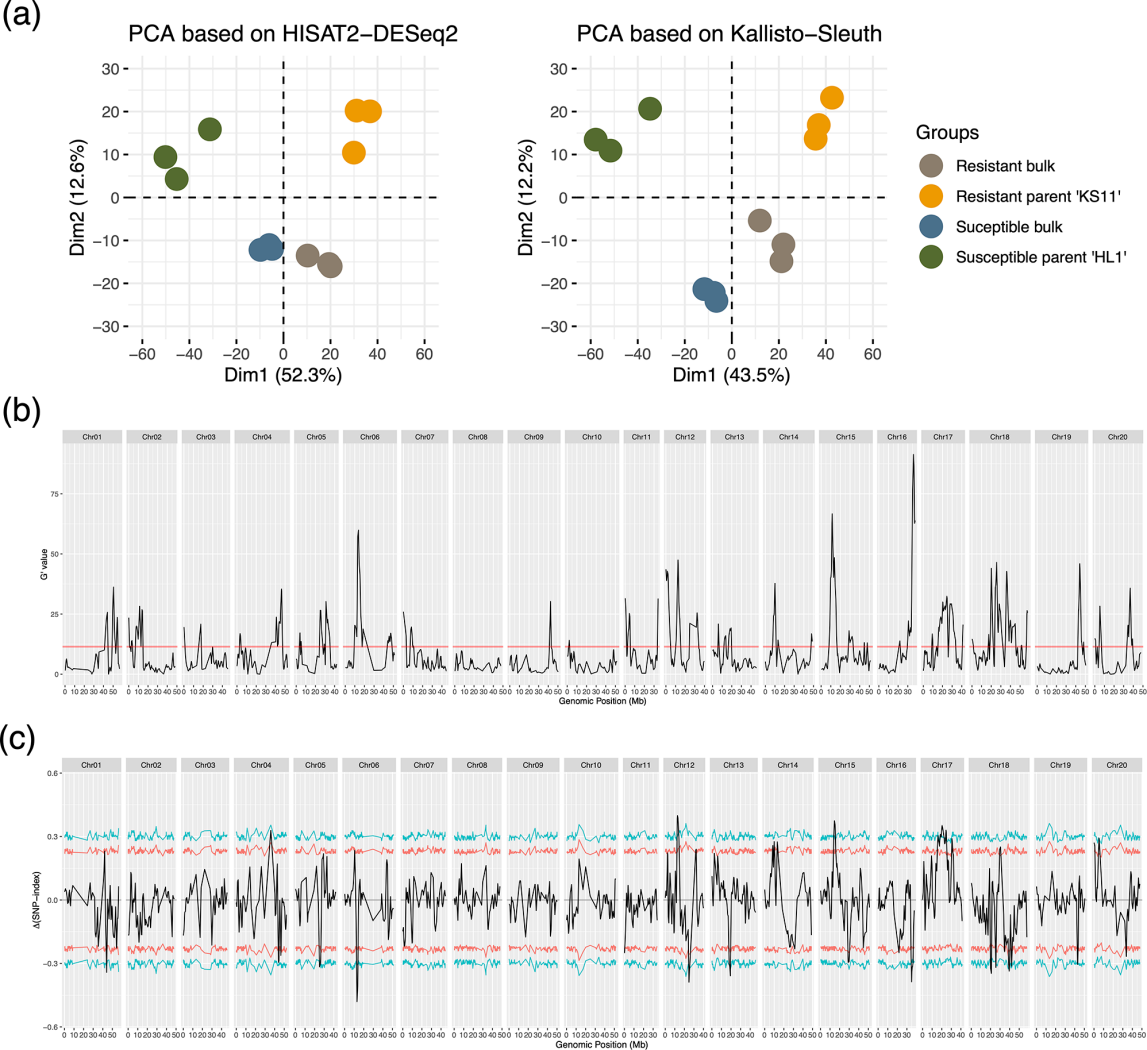
Principal component analysis (PCA) and bulk segregant analysis (BSA). (**a**) PCA based on the HISAT2-DESeq2 and Kallisto-Sleuth pipeline. Soybean PM resistance showed the major influence along the first dimension. (**b**) G' value, where the red line represents the false discovery rate at 0.01. (**c**) Δ(SNP-index), where blue line indicates the 99% confidence interval and red line indicates the 95% confidence interval

Each R and S bulk sample consisted of leaf RNA equally mixed from 10 F_2:3_ lineages to be one biological replicate. In total, the BSA comprised 30 resistant F_2:3_ lineages compared to 30 susceptible F_2:3_ lineages. The Illumina sequencing resulted in high qualified reads and mapping rates (Supplementary Material 2). The principal component analysis (PCA) based on HISAT2 and Kallisto both revealed a clear grouping for ‘KS11’, ‘HL1’, R bulk, and S bulk samples (Fig. 2A), and the PC1 using HISAT2 and Kallisto explained 52.3% and 43.5% of the variation, respectively, indicating the PM infection as a major factor in differentiating the sample grouping. Collectively, these results suggested a high quality and credibility of twelve RNA-Seq samples.

A total of 19,518 SNPs were identified across 20 Chrs. While G’ values defined significant 46 QTL (Table 1; Fig. 2B), the Δ(SNP-index) method only identified 10 QTL, a subset of all that being detected using the G’ values (Table 2). Notably, the regions on Chr06 (9.38 to 17.42 Mbp), Chr15 (8.16 to 15.97 Mbp), and Chr16 (29.54 to 37.68 Mbp) exhibited the top-three signal strengths according to the G’ statistics (Fig. 2C). Consequently, these three overlapping regions were subjected to downstream analyses.

**Table 1:** Significant QTL based on G’ value

QTL	Chr.	Start	End	Interval (Kbp)	SNPs in QTL	maxGprime	posMaxGprime	meanQval
1	Chr01	41,240,276	44,039,080	2,798.80	43	25.73	44,039,080	2.19E-03
2	Chr01	49,051,009	52,242,985	3,191.98	165	36.24	50,409,704	6.05E-04
3	Chr01	53,348,325	54,535,456	1,187.13	32	23.63	54,355,596	7.74E-04
4	Chr02	27,248	601,373	574.13	12	23.49	27,248	4.74E-04
5	Chr02	2,799,264	3,753,269	954.01	12	14.01	3,388,507	4.90E-03
6	Chr02	6,899,948	14,643,258	7743.31	218	28.26	11,326,683	6.47E-04
7	Chr03	115,054	1,081,719	966.67	50	19.56	115,054	1.75E-03
8	Chr04	2,936,165	4,080,540	1,144.38	42	16.87	3,253,769	1.19E-03
9	Chr04	35,995,923	47,428,227	11,432.30	162	35.41	46,239,500	1.12E-03
10	Chr05	3,271,448	4,279,942	1,008.49	53	17.17	3,965,942	3.28E-03
11*	**Chr05**	**25,031,503**	**27,363,173**	**2331.67**	**51**	**26.59**	**25,031,503**	**7.81E-04**
12	Chr05	30,454,125	35,160,277	4,706.15	290	30.22	30,962,290	9.00E-04
13*	**Chr06**	**9,379,113**	**17,419,325**	**8,040.21**	**287**	**59.91**	**13,782,174**	**2.67E-04**
14	Chr06	18,196,466	19,278,691	1,082.23	27	18.82	18,560,320	6.58E-04
15	Chr06	43,801,167	48,495,601	4,694.43	332	17.05	47,217,336	2.50E-03
16	Chr07	35,077	2,932,992	2,897.92	98	25.96	35,077	2.89E-04
17	Chr07	7,073,199	9,665,938	2,592.74	181	19.62	8,324,676	1.24E-03
18	Chr09	41,085,584	42,686,800	1,601.22	61	30.26	42,356,413	9.07E-04
19	Chr10	1,571,885	2,512,965	941.08	71	14.10	2,043,359	6.36E-03
20	Chr11	143,592	1,299,549	1,155.98	44	31.55	143,592	9.74E-05
21	Chr11	2,544,367	5,313,076	2,768.71	67	25.27	4,444,562	1.61E-03
22	Chr12	32,990	4,897,540	4,864.55	199	43.63	32,990	4.47E-04
23*	**Chr12**	**10,308,599**	**14,920,219**	**4,611.62**	**129**	**47.50**	**12,572,720**	**6.58E-04**
24	Chr12	24,784,017	33,230,491	8,446.47	32	25.51	32,399,508	8.71E-05
25	Chr13	12,113,411	14,044,703	1,931.29	36	16.00	13,660,348	2.06E-03
26*	**Chr13**	**16,613,274**	**19,313,959**	**2,700.69**	**61**	**19.26**	**19,313,959**	**2.04E-03**
27	Chr14	7,742,890	11,149,172	3,406.28	50	37.87	10,007,807	9.28E-04
28	Chr14	13,184,157	14,022,011	837.85	28	14.19	13,184,157	4.84E-03
29	Chr14	47,501,309	48,768,660	1,267.35	48	16.79	47,987,676	2.43E-03
30*	**Chr15**	**8,158,980**	**15,966,489**	**7,807.51**	**264**	**66.69**	**11,529,987**	**3.48E-04**
31	Chr15	28,958,496	33,111,213	4,152.72	10	18.07	28,958,496	2.34E-03
32	Chr15	47,894,172	48,151,989	257.82	19	13.03	47,967,989	7.89E-03
33*	**Chr16**	**29,537,679**	**37,675,335**	**8,137.66**	**359**	**91.40**	**35,913,263**	**3.79E-04**
34	Chr17	8,887,228	9,854,993	967.77	27	16.73	9,370,829	1.90E-03
35	Chr17	13,905,963	13,940,094	34.13	8	11.56	13,905,963	9.64E-03
36*	**Chr17**	**15,249,277**	**24,792,714**	**9,543.44**	**87**	**32.42**	**24,454,657**	**4.56E-04**
37	Chr17	26,551,382	33,488,594	6,937.21	119	29.33	27,919,226	1.04E-03
38	Chr17	40,588,752	41,302,057	713.31	32	20.64	41,302,057	1.83E-03
39	Chr18	156,869	1,536,235	1,379.37	166	14.59	156,869	3.88E-03
40	Chr18	24,255,171	30,561,365	6,306.19	26	46.51	25,654,977	1.33E-04
41*	**Chr18**	**34,518,559**	**45,178,142**	**10,659.58**	**116**	**42.77**	**35,960,162**	**7.62E-04**
42	Chr18	51,149,029	51,726,435	577.41	107	12.04	51,487,813	7.43E-03
43	Chr18	56,423,170	57,991,810	1,568.64	94	26.46	57,242,470	1.77E-03
44*	**Chr19**	**42,213,656**	**46,346,712**	**4,133.06**	**95**	**45.93**	**44,142,462**	**8.53E-04**
45	Chr19	47,903,184	48,412,831	509.65	12	13.34	48,171,387	6.21E-03
46	Chr20	33,702,942	39,259,901	5,556.96	263	35.78	36,740,673	5.03E-04

* QTL in bold were also identified by Δ(SNP-index).

**Table 2:** Significant QTL based on Δ(SNP-index)

QTL	Chr.	Start	End	Interval (Kbp)	SNPs in QTL	Peak of DeltaSNP	Pos of QTL Peak	avgDeltaSNP
1	Chr05	25,031,503	25,829,235	797.73	16	-0.32	25,031,503	-0.31
2	Chr06	12,772,969	13,997,351	1224.38	47	-0.48	12,990,670	-0.44
3	Chr12	12,572,720	13,457,877	885.16	13	0.40	12,833,468	0.39
4	Chr13	18,126,034	19,313,959	1187.93	11	-0.36	19,313,959	-0.33
5	Chr15	13,972,273	15,675,119	1702.85	101	0.37	14,625,059	0.35
6	Chr16	34,955,172	36,416,266	1461.09	62	-0.39	34,955,172	-0.32
**7**	Chr17	18,264,002	18,708,771	444.77	19	0.31	18,708,771	0.30
8	Chr17	20,208,120	20,926,849	718.73	11	0.35	20,390,168	0.35
9	Chr18	39,638,714	39,987,036	348.32	14	-0.34	39,638,714	-0.33
10	Chr19	42,855,894	42,957,442	101.55	17	-0.30	42,957,442	-0.30

### Differential expression analysis between R and S bulks

**Fig. 3:**
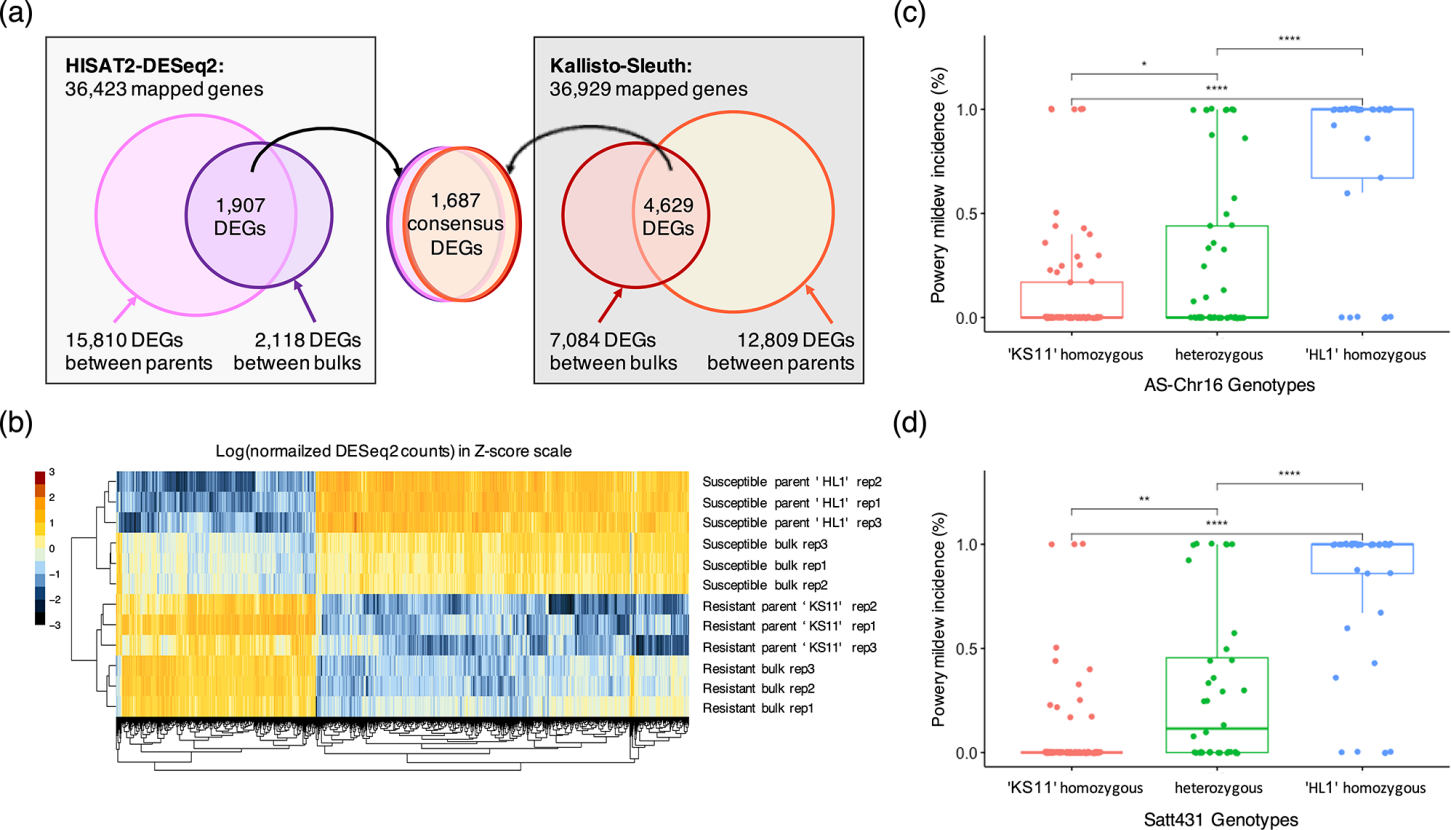
Differential expression analysis. (**a**) Venn diagram showing the consensus 1,687 DEGs identified by comparing the resistant and susceptible parents as well as bulks using both HISAT2-DESeq2 and Kallisto-Sleuth pipeline. (**b**) Heatmap of the consensus 1,687 DEGs represented by DESeq2 quantification. (**c**) Mean separation test for PM incidence in the F_2_ generation according to the AS-Chr16 genotype, and (**d**) the Satt431 genotype. The Kruskal-Wallis test and the Dunn’s test was applied to determine significant difference at α = 0.01

Using the HISAT2-DESeq2 pipeline, there were 15,810 DEGs found between the comparison of ‘KS11’ and ‘HL1’ parents and 2,118 DEGs found between the comparison of R bulks and S bulks. A total of 1,907 DEGs shared by the parental and bulk comparison were kept for downstream analysis. On the other hand, using the Kallisto-Sleuth pipeline, there were 12,809 DEGs found between the comparison of ‘KS11’ and ‘HL1’ parents and 7,084 DEGs found between the comparison of R bulks and S bulks (Fig. 3A). A total of 4,629 DEGs shared by the parental and bulk comparison were kept for downstream analysis. In comparing 1,907 and 4,629 DEGs identified by these two pipelines, there were 1,687 DEGs agreed in both methods, and the Pearson’s correlation revealed a R^2^ value of 0.98 for these 1,687 DEGs (Fig. 3B, C). Accordingly, these consensus DEGs were considered in this study. Among these DEGs, 35, 25, and 49 genes were found in the significant QTL region on Chr06, Chr15, and Chr16 identified from BSA, respectively (Supplementary Material 2).

Subsequently, the significant SNPs nearby the DEG showing a strong differential expression were selected for designing AS-primers to validate the mapping credibility based on the accuracy of phenotypic prediction in the F_2_ population. These AS-primers include AS-Chr06, which distinguishes a TTG/T indel polymorphism, locates on the 13,445,340 bp nearby the Glyma.06G162400 (Supplementary Material 3). Another AS-Chr15, which differentiates a A/T polymorphism, locates on the 15,275,091 bp nearby the Glyma.15G170500 (Supplementary Material 3). Unfortunately, phenotypic mean separation using AS-Chr06 and AS-Chr15 failed to discriminate resistant and susceptible F_2_ progenies (Supplementary Material 3); therefore, these two peaks may be false positive signals. On the other hand, AS-Chr16, which differentiates a T/C polymorphism, locates on the 35,712,161 bp nearby the Glyma.16G195600. According to the genotypes defined by AS-Chr16, the Kruskal-Wallis and Dunn’s test detected significant difference among the homozygous allele 1 (‘KS11’), the heterozygous allele, and the homozygous allele 2 (‘HL1’), where the progenies with homozygous allele 2 exhibited the highest disease incidence (Fig. 3C). In addition, phenotypic prediction using AS-Chr16 reached 83% accuracy in discriminating the F_2_ progenies exhibiting resistance and susceptibility. Similar results in terms of additive effect and prediction accuracy can be observed using the Satt431 marker for genotyping (Fig. 3D). The results indicated the peak identified on Chr16 contributes to the PM resistance derived from ‘KS11’.

### Linkage disequilibrium (LD) analysis on Chr16

**Fig. 4:**
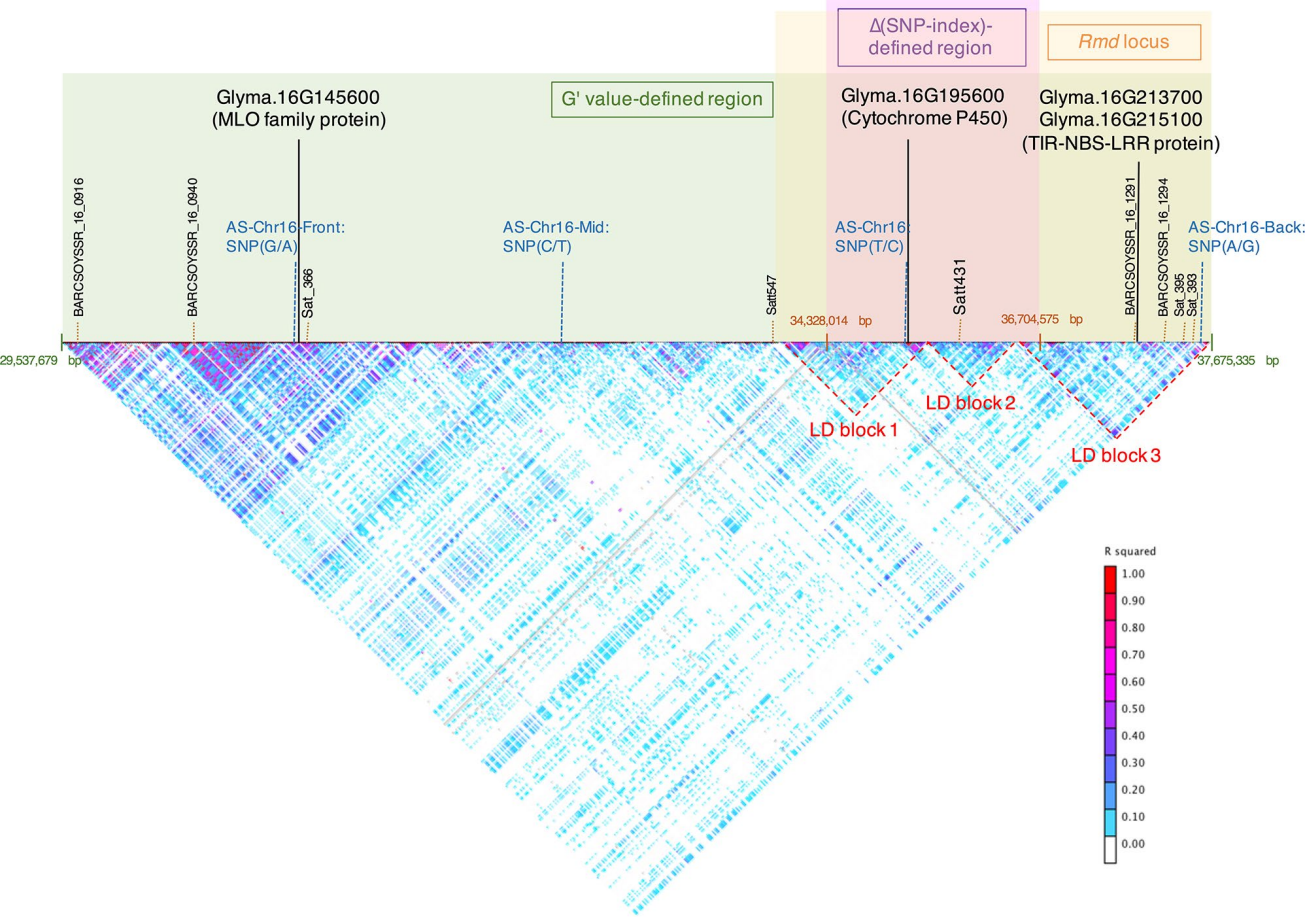
Linkage disequilibrium (LD) analysis for the *Rmd* locus on the Chr16. The green background highlights the G’ value-defined region, the purple background highlights the Δ(SNP-index)-defined region, and the yellow background highlights the *Rmd* locus based on previous literatures. Glyma.16G145600, which is an MLO family protein, locates within the G’ value-defined region. However, Glyma.16G145600 locates in a separate LD block from *Rmd* locus according to the guide of USDA SoySNP50K data, and the AS-Chr16-Mid primers confirmed the independent segregation to AS-Chr16 primers and Satt431. Meanwhile, there are three LD blocks within the *Rmd* locus, where the GmRmd1 (Glyma.16G214200) locates in the LD block 3. The AS-Chr16 primers were not segregated from Satt431 or AS-Chr16-Back in the population, therefore, the Δ(SNP-index)-defined region derived from ‘KS11’ is overlapped with the *Rmd* locus from previous literatures. Solid black lines indicated significant DEGs identified in this study, for which the expressions of Glyma.16G145600 and Glyma.16G195600 were higher in the susceptible lines, and Glyma.16G214300 and Glyma.16G214500 were higher in the resistant lines

Focusing on the *Rmd* locus reported in literature and identified in this study, G' value identified a broader region than Δ(SNP-index) (Fig. 4). Surprisingly, the G’ value-defined region harbors an MLO gene (Glyma.16G145600). Since MLO genes are well known to involve in PM resistance in many plant hosts, and the mutation in MLO contributes the *loss-of-susceptibility* mechanism for plants to become PM resistance [41], the observation on the lower gene expression of Glyma.16G145600 in the R parent and R bulk samples may be one of the possible resistance sources derived from ‘KS11’. Based on the guide of USDA SoySNP50K data which harbors historical recombination events, the location of Glyma.16G145600 might be segregated from the Δ(SNP-index)-defined region. In order to confirm the linkage, the AS-Chr16-Front and AS-Chr16-Mid primers were used to genotype the F_2_ population, and the results suggested the linkage has not been broken down in the F_2_ generation compared to Satt431 (${\chi }^{2} \text{t}\text{e}\text{s}\text{t}\text{s}$, *p* < 0.01). In summary, the analyses on LD and SNPs suggested the extended region defined by G’ value, which includes the MLO family gene Glyma.16G145600, may be associated with the PM resistance from ‘KS11’.

On the other hand, the Δ(SNP-index)-defined region mainly include two LD blocks, where the AS-Chr16 was designed in the first LD block and the Satt431 located in the second LD block (Fig. 4). As the phenotypic prediction accuracies by AS-Chr16 and Satt431 were close and the independence test of AS-Chr16 and Satt431 was significant (${\chi }^{2} \text{t}\text{e}\text{s}\text{t}$, *p* < 0.01), the results suggested that these LD regions (specifically, the region from AS-Chr16 to Satt431) were not segregated in the F_2_ population. However, the Δ(SNP-index)-defined region does not harbor any Leucine-rich repeat (LRR) resistance genes as reported in other PM resistance studies. Instead, many LRR resistance genes locate in the later region of the Δ(SNP-index)-defined region. In order to confirm whether the later region was segregated from the Δ(SNP-index)-defined region, several markers were selected to genotype the F_2_ population. Because most SSR markers were not polymorphic between the ‘KS11’ and ‘HL1’, the AS-Chr16-Back primers were designed to genotype the F_2_ population. The independence test was significant (${\chi }^{2} \text{t}\text{e}\text{s}\text{t}\text{s}$, *p* < 0.01), indicating the Δ(SNP-index)-defined region does not segregated yet from the location harboring candidate resistance genes mostly from Glyma.16G205100 to Glyma.16G215100.

Although the PM resistance source derived from ‘KS11’ overlaps with the *Rmd* locus reported in other literatures, the up-regulated DEGs appeared unique for ‘KS11’. Candidate resistance genes such as Glyma.16G213900, Glyma.16G214200, Glyma.16G214800, Glyma.16G214900, Glyma.16G215200, and Glyma.16G215300 from previous literatures [42, 43] were not up-regulated in this study. The only up-regulated DEGs that encode TIR-NBS-LRR proteins identified in ‘KS11’ were Glyma.16G213700 and Glyma.16G215100. While up-regulated DEGs in previous studies were mostly found at time points within 48-hours post infection, our sampling stage at around the soybean R2 growth stage, which may introduce expression difference in comparison. Future researches on the functional validation for Glyma.16G213700 and Glyma.16G215100 in soybean PM resistance may further uncover their contributions in mechanism.

## Discussion

The initial documentation of soybean PM resistance can be attributed to Grau and Laurence [44]. Their work identified two types of resistance. The first type, referred to as *Rmd*, exhibited adult-plant resistance and was observed in cultivars such as ‘Chippewa 64’, ‘Blackhawk’, and ‘Williams’ [45, 46]. The second type, named *Rmd-c*, demonstrated life-span resistance and was found in cultivars like ‘CNS’ and ‘Wilkin’ [44, 47, 48]. It has been suggested that *Rmd* and *Rmd-c* are allelic and exhibit dominant inheritance [45]. Planting cultivars with *Rmd* or *Rmd-c* has been shown to provide approximately 18% higher yields compared to the susceptible genotype *rmd* in susceptible cultivars. Additionally, the life-span resistance of *Rmd-c* has been associated with an additional 7% yield improvement compared to the adult-plant resistance of *Rmd* [49]. Therefore, incorporating PM resistance into soybean breeding has proven effective, as observed in PM of various other crops [50].

The mapping of the *Rmd* locus did not occur until 1994, and it was discovered on the J linkage group (Chr16), adjacent to the *Phytophthora sojae* resistance locus *Rps2* and the *Bradyrhizobium* nodulation locus *Rj2* [51]. Subsequent studies independently mapped PM resistance derived from different resistant donors, consistently identifying the same locus on Chr16 with slight variations in the interval. For instance, Kang and Mian (2010) [52] employed SSR markers and BSA to analyze a population derived from the season-long resistant germplasm PI 243,540. They identified the interval between Satt_224 and Sat_393 on Chr16, with BARC-021875-04228 being the closest SSR marker. The PM resistance locus PMD_PI 567301B was identified through BSA on a population derived from the resistant donor PI 567301B. The locus was located between SSR markers Satt431 and Sat_394, and subsequent investigation narrowed it down to a smaller region containing four candidate genes (Glyma16g34070, Glyma16g34090, Glyma16g34110, and Glyma16g34120) between BARCSOYSSR_16_1291 to BARCSOYSSR_16_1298 [53]. Another study focused on the F_2:3_ population derived from the resistant parent V97-3000 also identified a locus on Chr16, with the interval being defined by Satt547 and Sat_393. In this case, three candidate genes (Glyma16g34030, Glyma16g34070, and Glyma16g34090) were proposed [54].

The most recent investigation on soybean PM resistance focused on the cultivar ‘B13’. Through BSA on an F_2_ population and subsequent fine mapping on an F_8_ population, Jiang et al. (2019) [42] identified a Mendelian locus on Chr16 with a genomic region of 188 Kb, housing 17 candidate genes associated with resistance. Expanding upon this, a comprehensive analysis integrating genome-wide association studies of PM resistance in 467 soybean accessions and comparisons of *de novo* genomes revealed that the mechanism underlying *GmRmd1* may involve a presence/absence variation of the Glyma.16G214200 gene. The contribution of Glyma.16G214200 to PM resistance was further validated through the application of CRISPR-Cas9 knockout approach. However, it should be noted that among 2,141 PM-resistant accessions examined, only 1,018 accessions were found to carry the *GmRmd1* gene, suggesting the presence of additional PM resistance genes in different genetic backgrounds [43]. In another study focused on the resistant cultivar ‘Zhonghuang 24’, the adult plant resistance on Chr16 was also identified. The interval was subsequently fine-mapped to the region encompassing Glyma.16G214300 to Glyma.16G214700. Expression analysis indicated that Glyma.16G214300 and Glyma.16G214500 were up-regulated in response to PM infection. Additionally, through F_2_ segregation tests on the crossing population of ‘Zhonghuang 24’ and ‘B13’, it was suggested that ‘Zhonghuang 24’ may carry a distinct but closely linked source of resistance compared to ‘B13’ [55]. Accordingly, although studies have identified the *Rmd* region on Chr16 as the primary resistance source for soybean PM, allelic diversity has been observed in different genetic backgrounds and ‘KS11’ may harbor different candidate genes or regulations to confer PM resistance.

In addition to LRR genes, PM resistance can be achieved by *loss-of-susceptibility* on genes such as MLO genes to confer PM resistance in many plants and STAY-GREEN genes to confer foliar chlorosis of soybean sudden death syndrome [10, 41]. Focusing the plant MLO genes, which can be dated back to the earliest documentation in 1942 [56], MLO genes encode plasma-membrane localized proteins that interact with syntaxin proteins (encoded by the PEN1 genes) to modulate vesicle trafficking on the fungal penetration sites. It has been suggested that PEN1/2/3 regulates cell walls and/or deliver phytoalexins to defend against non-adaptive fungus [57]. However, adaptive PM fungi may have evolved to utilize the wild type MLO-PEN1 mechanism for haustoria development. When mutations happen on the MLO gene, *loss-of-susceptibility* occurs to confer the recessive-inherited *mlo* genotype and durable PM resistance [58]. Nonetheless, it has been found that *loss-of-susceptibility* mechanism such as *mlo* and *stay-green* genotypes come with physiological penalties such as necrotic spots [10, 41]. Therefore, the implementation of weak mutation with fewer physiological disadvantages would be the key to providing applicable PM resistance in plant breeding [59]. While *mlo* resistance has been documented in dozens of plant species, the *mlo* genotype has not been found in soybean to confer PM resistance even though 20 MLO genes have been found in the soybean genome [60]. It was a surprise that the G’ value-defined region on Chr16 harbors a soybean MLO gene (Glyma.16G145600), an ortholog to Arabidopsis MLO12. The expression of Glyma.16G145600 was found about 40-folds and 8-folds higher in the susceptible parent and bulks, respectively (Table S2). As a previous study suggested about a 10-fold difference between the susceptible MLO genotype and resistant *mlo* genotype [61], our study highlighted the question of whether Glyma.16G145600 contributes a minor effect in PM resistance governed by the *Rmd* locus. However, the Arabidopsis *mlo12* genotype (At2g39200) was found to contribute none in PM resistance [62], and the PM resistance derived from ‘KS11’ is a dominant trait. Therefore, it is also likely that limited recombination events in the F_2_ generation constrained the mapping resolution to segregate Glyma.16G145600 from the *Rmd* locus. Future fine mapping using higher generations or reverse genetics approaches may provide detailed insight into the possibility of the soybean MLO gene participating in the PM resistance.

## Conclusions

This study demonstrated that BSR-Seq is a robust tool to identify loci associated with soybean traits such as disease resistance. The integration of genotypes and gene expressions between the resistant bulk and the susceptible bulk, together with the phenotypic prediction using allele-specific primers (AS-primers), confirmed the presence of a locus on Chr16 harboring both the *Rmd* locus with unique up-regulated LRR genes (Glyma.16G213700 and Glyma.16G215100) and a down-regulated MLO gene (Glyma.16G145600). Collectively, future studies utilizing BSR-Seq, AS-primers, and the *Rmd* locus together with the MLO gene may provide further insights to improve soybean genetics and breeding for PM resistance.

## Methods

### Plant materials and bulking for PM resistance

The parental varieties were the PM-resistant edamame variety ‘Kaohsiung 11’ (‘KS11’) [63] and the PM-susceptible soybean variety ‘Hualien 1’ (‘HL1’) (Tsai CW. 1979). A crossing population was generated, and the F_1_, F_2_, and F_2:3_ progeny populations were maintained in the greenhouse at Taoyuan District Agricultural Research and Extension Station. Two parents were crossed in August 2018, and the F_1_ and F_2_ populations were propagated in March 2019 and March 2020, respectively. Twenty F_1_ seeds from different crossings were planted to obtain F_2_ seeds, and 249 F_2_ seeds were planted to obtain F_2:3_ seeds.

The F_2:3_ population was planted in February 2021 to favor the occurrence of soybean PM in the spring weather conditions. The susceptible parent ‘HL1’ was randomly planted in the greenhouse to ensure the natural PM inoculum uniformly cover the whole planting area, and all susceptible parent ‘HL1’ were confirmed to shown PM symptoms. The F_2:3_ seeds were classified as either presence or absence of PM symptoms on soybean leaves. The frequency of PM incidence of each F_2_ lineage ($\frac{\text{n}\text{u}\text{m}\text{b}\text{e}\text{r} \text{o}\text{f} \text{P}\text{M}-\text{i}\text{n}\text{f}\text{e}\text{c}\text{t}\text{e}\text{d} {\text{F}}_{2:3} \text{p}\text{l}\text{a}\text{n}\text{t}\text{s} }{\text{n}\text{u}\text{m}\text{b}\text{e}\text{r} \text{o}\text{f} {\text{F}}_{2:3} \text{p}\text{l}\text{a}\text{n}\text{t}\text{s} \text{i}\text{n} \text{t}\text{h}\text{e} \text{s}\text{a}\text{m}\text{e} {\text{F}}_{2} \text{l}\text{i}\text{n}\text{e}\text{a}\text{g}\text{e}}$) was used to assess the homozygosity or heterozygosity status for each F_2_ lineage. F_2_ lineages with 0% or 100% PM incidence were considered as homogenous resistance and susceptibility, respectively (Supplementary Material 4). The resistant bulk (R bulk) and susceptible bulk (S bulk) were selected from lineages with homogenous resistance and susceptibility, respectively. Leaf samples were collected from the resistant ‘KS11’ (R parent), susceptible ‘HL1’ (S parent), R bulk, and S bulk in the greenhouse. The leaf surfaces were rinsed with water to remove dusts, and immediately stored with dry ice for transfer to the laboratory for RNA extraction.

### RNA extraction and sequencing

RNA was extracted using the TRIzol™ Reagent (Invitrogen™, Thermo Fisher Scientific, Waltham, MA, USA) following a protocol involving chloroform extraction, isopropanol precipitation, and LiCl_2_ purification. The RNA samples were quantified using the NanoDrop One (Thermo Fisher Scientific) and Qubit™ Fluorometer (Invitrogen™) and subjected to quality control through electrophoresis and integrity analyzer QSep 100 (Bioptic, New Taipei City, Taiwan). For the R or S parent samples, leaves were collected from three independent plants of ‘KS11’ or ‘HL1’, respectively. An equal amount of purified RNA from the three R or S parent samples was mixed evenly to represent a biological replicate. As for the R or S bulk samples, in order to avoid sampling heterozygous F_2_ lineages into two extreme bulks, the F_2_ lineages were collected into a R bulk only when a minimum of 10 F_2:3_ plants all free from PM infection. Because there was less number of susceptible F_2_ lineages to be selected, the F_2_ lineages were collected when at least 6 F_2:3_ plants all exhibited PM infection. An equal amount of purified RNA from ten F_2:3_ plants exhibiting homogenous resistance or susceptibility was mixed evenly to represent a biological replicate (Fig. 1). Three biological replicates were prepared for R parent, S parent, R bulk, and S bulk, resulting in a total of 12 samples. These samples were subjected to RNA-Seq analysis using the Illumina NovaSeq platform with paired-end 150 bp (Biotools, New Taipei City, Taiwan).

### Bulk segregant analysis (BSA) and linkage disequilibrium (LD)

The sequencing data were quality assessed using FastQC and Cutadapt version 2.3 to trim the adaptors and low quality reads below Q30, before subjected to alignment against the ‘Williams 82’ reference genome a2.v1 using BWA version 0.7.17 [64]. Subsequently, SAMtools version 1.13 was utilized to convert the sam files into bam files, as well as to sort and identify duplicated reads within the bam files. The bam files were reformatted using Picard version 2.26.0 [65] and served as input for variant calling in GATK version 3.8.1.0 [66, 67]. GATK version 4.2.3.0 was further utilized to eliminate variants with a quality score below 20 and generate a vcf file after sorting with Picard.

The vcf file was employed as input in the R package “QTLseqr” version 0.7.5.2 for subsequent analysis [68]. The default SNP filtering thresholds were utilized, including a minimum total depth of 100, maximum total depth of 400, depth difference of 100, minimum sample depth of 40, reference allele frequency of 0.3, and minimum GQ of 99. To perform the QTL analysis, the window size was set at 1 Mbp, population structure was defined as F_2_, bulk size was set at 30, and the analysis was replicated 10,000 times [69]. The significance of ΔSNP-index was determined at a 99% confidence interval and at least 10 SNPs within QTL. The significance of G’ statistics was determined at *q* values below 0.01 and at least 10 SNPs within QTL. For the top-three significant QTL located on Chr 6, 15, and 16, local LD analysis was assessed using the SoySNP50K dataset [70] in TASSEL 5 for this analysis [71].

### Differential expression analysis

The RNA-Seq data were mapped to the ‘Williams 82’ a2v1 genome using HISAT2 version 2.2.1 [72]. Subsequently, SAMtools and the featurecounts function of Subread version 2.0.0 were employed to reformat the data and quantify gene expressions [73]. The quantified results were then analyzed using the R package “DESeq2” version 1.38.1 to obtain higher confidence in gene expression analysis [74], and only genes with a minimum count of 1 in more than three samples were retained for downstream analysis. The Benjamini-Hochberg adjusted *p*-value below 0.05 to determine significance. Meanwhile, the k-mer-based pseudoalignment approach utilizing Kallisto version 0.46.2 and Sleuth version 0.30 was applied and significance was determined at q-value below 0.05 [75, 76]. The Pearson’s correlation of significant differentially expressed genes (DEGs) resulted from HISAT2-DESeq2 and Kallisto-Sleuth were estimated, and the consensus DEGs were considered as candidate genes.

### Phenotypic prediction by allele-specific primers (AS-primers) in F_2_ population

The significant SNPs on Chr 6, 15, and 16 most nearby the DEGs were selected for designing AS-primers, which included the AS-Chr06 primers (F’-FAM: GAA GGT GAC CAA GTT CAT GCT TGG AAA GGT AGC TAG GCc TTG; F’-HEX: GAA GGT CGG AGT CAA CGG ATT TGG AAA GGT AGC TAG GCA TTA, R’: GGA GAC TCG AGT GTT TGA GC), AS-Chr15 primers (F’-FAM: GAA GGT GAC CAA GTT CAT GCT TCT TTG CCC ACT TGT gGG CT; F’-HEX: GAA GGT CGG AGT CAA CGG ATT TCT TTG CCC ACT TGT TGt CA, R’: CAC TGC AGT TGA ATT TAT TAC), AS-Chr16-front primer (F’-FAM: GAA GGT GAC CAA GTT CAT GCT CGT TTT GTC CCT GAc AAG; F’-HEX: GAA GGT CGG AGT CAA CGG ATT CGT TTT GTC CCT GAc AAA, R’: CTG CTT TGC TAT TGA TCA GG), AS-Chr16-mid primer (F’-FAM: GAA GGT GAC CAA GTT CAT GCT GTG GAT GAT GAG TCA GTT cAC; F’-HEX: GAA GGT CGG AGT CAA CGG ATT GTG GAT GAT GAG TCA GTT cAT, R’: CTG TGT CAT CCT TTG GAA TGC C), AS-Chr16 primers (F’-FAM: GAA GGT GAC CAA GTT CAT GCT GGT TTG TTT TCG GCA gCT TT; F’-HEX: GAA GGT CGG AGT CAA CGG ATT GGT TTG TTT TCG GCA TCa TC, R’: AGT TTG GGA TAT TTC CTC CC), and AS-Chr16-back primer (F’-FAM: GAA GGT GAC CAA GTT CAT GCT ATT CCC TCT GTT CCC ACg CA; F’-HEX: GAA GGT CGG AGT CAA CGG ATT ATT CCC TCT GTT CCC ACg CG, R’: GTC GTT TAT GAC GGA GAT GTC GG) where the lowercase letters indicate intentional mismatches in design to increase specificity and underlines indicate the consensus sequences to universal probes [77]. The FAM- or HEX-labelled universal probes (5’-FAM: AGC GAT GCG TTC GAG CAT CGC T*GA AGG TGA CCA AGT TCA TGC T, 5’-HEX: AGG ACG CTG AGA TGC GTC CT*G AAG GTC GGA GTC AAC GGA TT) were adapted from previous Amplifluor fluorescenct probes, where the asterisks highlight the BHQ1-labeled nucleotides and underlines indicate the consensus sequences to AS-primers [78, 79]. For the AS-primers, the touchdown thermocycling condition was optimized for the Tools Easy 2xProbe qPCR Mix (BioTools) and validated by gel electrophoresis to ensure the forward primers recognize the parental alleles, respectively. The thermycycling condition included: an initial denaturing for 180 s at 95 °C; a 10-cycle touchdown PCR include a denaturing for 20 s at 95 °C, an annealing for 15 s from 77 °C down to 65 °C by decreasing 1.2 °C each cycle, and an extending for 10 s at 72 °C; lastly, a 32-cycle two-step PCR include a denaturing for 20 s at 95 °C, and an extending for 40 s at 55 °C. The PCR was conducted on the BioRad CFX Connect Real-Time PCR Detection System (Hercules, CA, USA).

In addition, the SSR markers BARCSOYSSR_16_0878, BARCSOYSSR_16_0916, BARCSOYSSR_16_0940, BARCSOYSSR_16_1291, BARCSOYSSR_16_1294, Sat_093, Sat_366, Sat_393, Satt395, Satt215, Satt431, Satt547, Satt622, Satt712 were evaluated for the polymorphisms between ‘KS11’ and ‘HL1’, and only the polymorphism Satt431 was included as a comparison to the AS-Chr16 primers. All the leaf DNA was extracted from the F_2_ soybean samples using the Plant Genomic DNA Mini Kit (Geneaid, New Taipei City, Taiwan). Phenotyppic mean separation by genotypes (SNP or SSR) were conducted in R environment v4.3.0. The Kruskal-Wallis test and the Dunn’s test was applied to determine significant difference at α = 0.01.

### Electronic supplementary material

Below is the link to the electronic supplementary material.

Supplementary Material 1

Supplementary Material 2

Supplementary Material 3

Supplementary Material 4

Supplementary Material 5

## Data Availability

The RNA-Seq data were deposited in the NCBI BioProject PRJNA987889. (According to the NCBI depository option, the data will be publicly available upon the release date or the manuscript acceptance date, whichever comes the first.)
