# Supplementary material for: Application of bulk segregant RNA-Seq (BSR-Seq) and allele-specific primers to study soybean powdery mildew resistance
Source: BMC Plant Biol. 2024 Mar 1;24:155. doi: 10.1186/s12870-024-04822-1 (PMC10905810; doi:10.1186/s12870-024-04822-1)
Supplement: Supplementary file 4 — Supplementary Material 4 [file 12870_2024_4822_MOESM4_ESM.docx]

**Support Information**

**TABLE S1.** Metadata of 'Kaohsiung 11' x 'Huanlien 1' population

**TABLE S2.** Mapping statistics of 12 RNA-Seq samples.

**TABLE S3.** The consensus 1,687 differentially expressed genes identified by HISAT2-DESeq2 and Kallisto-Sleuth.


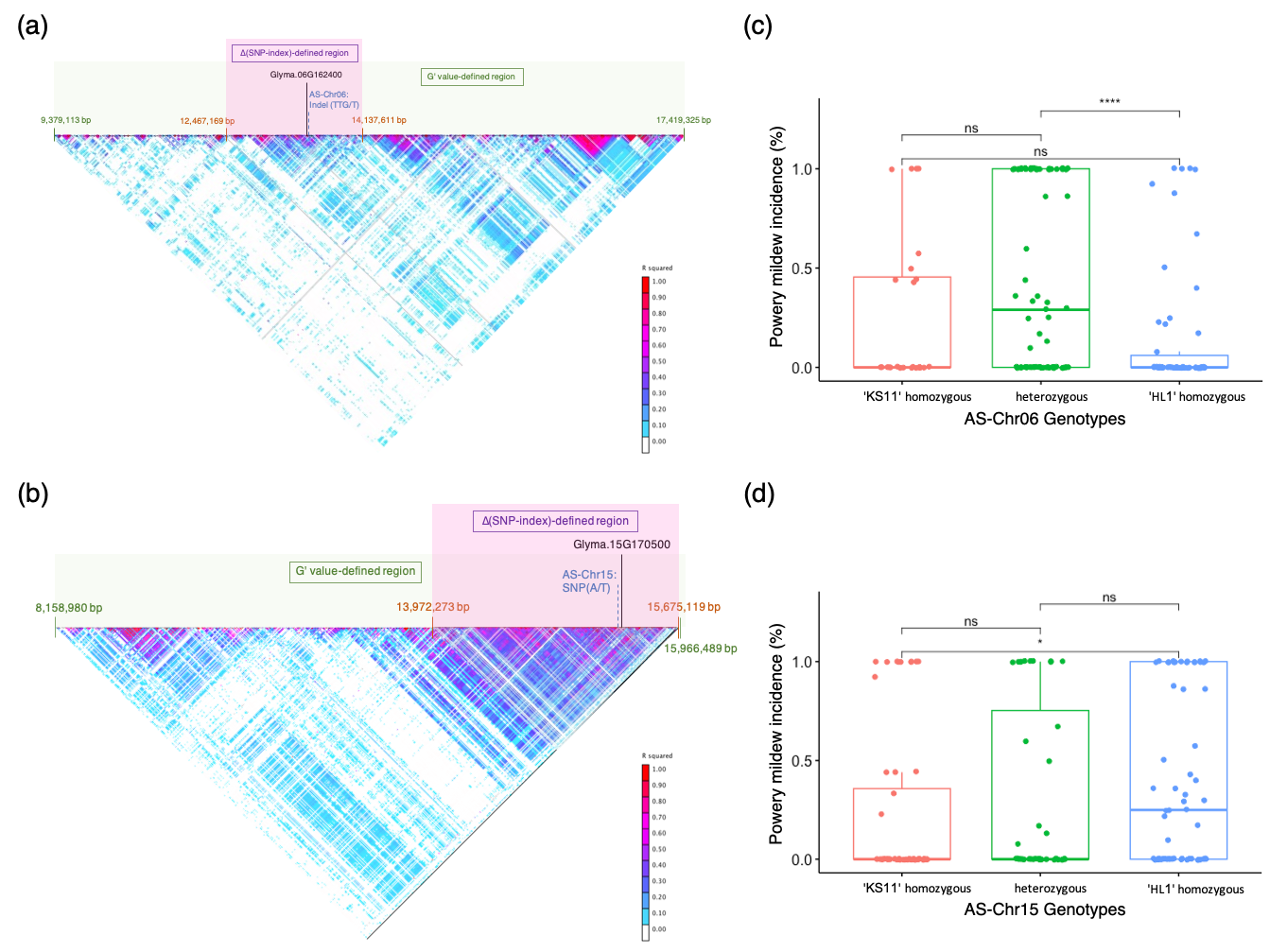


**Figure S1.** LD analyses and mean separation by genotypes detected using the AS-primers. (a) Candidate locus on Chr06 defined by G' and Δ(SNP-index). AS-Chr06 was designed nearby the most significant differentially expressed gene (DEG), Glyma.06G162400, in this region. (b) Candidate locus on Chr15 defined by G' and Δ(SNP-index). AS-Chr15 was designed nearby the most significant DEG, Glyma.15G170500, in this region. (c) Mean separation on the powdery mildew (PM) incidence by AS-Chr06 genotypes. (d) Mean separation on the PM incidence by AS-Chr15 genotypes. The Kruskal-Wallis test and the Dunn’s test was applied to determine significant difference at α = 0.01.
